# Dopamine- and Grape-Seed-Extract-Loaded Solid Lipid Nanoparticles: Interaction Studies between Particles and Differentiated SH-SY5Y Neuronal Cell Model of Parkinson’s Disease

**DOI:** 10.3390/molecules29081774

**Published:** 2024-04-13

**Authors:** Rosanna Mallamaci, Debora Musarò, Marco Greco, Antonello Caponio, Stefano Castellani, Anas Munir, Lorenzo Guerra, Marina Damato, Giuseppe Fracchiolla, Chiara Coppola, Rosa Angela Cardone, Mehdi Rashidi, Roberta Tardugno, Sara Sergio, Adriana Trapani, Michele Maffia

**Affiliations:** 1 Department of Biosciences, Biotechnologies and Environment, University of Bari “Aldo Moro”, 70125 Bari, Italy; rosanna.mallamaci@uniba.it (R.M.); lorenzo.guerra1@uniba.it (L.G.); rosaangela.cardone@uniba.it (R.A.C.); 2 Department of Biological and Environmental Science and Technology, University of Salento, Via Lecce—Monteroni, 73100 Lecce, Italy; debora.musaro@unisalento.it (D.M.); sara.sergio@unisalento.it (S.S.); 3 Department of Pharmacy-Drug Sciences, University of Bari “Aldo Moro”, Via Orabona 4, 70125 Bari, Italy or antonello.caponio@uniba.it (A.C.); giuseppe.fracchiolla@uniba.it (G.F.); roberta.tardugno@uniba.it (R.T.); 4 Department of Precision and Regenerative Medicine and Ionian Area (DiMePRe-J), University of Bari “Aldo Moro”, 70125 Bari, Italy; stefano.castellani@uniba.it; 5 Department of Mathematics and Physics “E. De Giorgi”, University of Salento, Via Lecce—Arnesano, 73100 Lecce, Italychiara.coppola@unisalento.it (C.C.); mehdi.rashidi@unisalento.it (M.R.); 6 Department of Experimental Medicine, University of Salento, Via Lecce—Monteroni, 73100 Lecce, Italy; marina.damato@unisalento.it

**Keywords:** Parkinson’s disease, dopamine, solid lipid nanoparticles, grape seed extract, alpha-synuclein, oxidative stress

## Abstract

Parkinson’s disease (PD) is a prevalent neurodegenerative disorder, primarily associated with dopaminergic neuron depletion in the Substantia Nigra. Current treatment focuses on compensating for dopamine (DA) deficiency, but the blood–brain barrier (BBB) poses challenges for effective drug delivery. Using differentiated SH-SY5Y cells, we investigated the co-administration of DA and the antioxidant Grape Seed Extract (GSE) to study the cytobiocompability, the cytoprotection against the neurotoxin Rotenone, and their antioxidant effects. For this purpose, two solid lipid nanoparticle (SLN) formulations, DA-co-GSE-SLNs and GSE-ads-DA-SLNs, were synthesized. Such SLNs showed mean particle sizes in the range of 187–297 nm, zeta potential values in the range of −4.1–−9.7 mV, and DA association efficiencies ranging from 35 to 82%, according to the formulation examined. The results showed that DA/GSE-SLNs did not alter cell viability and had a cytoprotective effect against Rotenone-induced toxicity and oxidative stress. In addition, this study also focused on the evaluation of Alpha-synuclein (aS) levels; SLNs showed the potential to modulate the Rotenone-mediated increase in aS levels. In conclusion, our study investigated the potential of SLNs as a delivery system for addressing PD, also representing a promising approach for enhanced delivery of pharmaceutical and antioxidant molecules across the BBB.

## 1. Introduction

Parkinson’s disease (PD) is the second-most common late-onset neurodegenerative disease, characterized by a series of common motor symptoms, such as tremor at rest, akinesia, rigidity, and postural instability. In addition, PD is associated with a plethora of non-motor symptoms like autonomic dysfunctions, sleep disturbances, cognitive alterations, and mood disorders [1].

Characteristically, the appearance of the first overt clinical symptoms is related to the depletion of the dopaminergic neuronal population residing in the mesencephalic Substantia Nigra (SN) that results in a significant drop in the neurotransmitter dopamine (DA) levels in the thalamus [2]. The reduced ability to generate DA is primarily associated, although not exclusively, with the motor symptoms and loss of coordination in PD [3].

The exact cause of the cell loss in the Central Nervous System (CNS) is not fully understood, although some environmental, lifestyle, and genetic factors, as well as age and inflammatory conditions, have been shown to play a role in PD development and progression [4,5].

The treatment of the disease is primarily symptomatic and aims to compensate for the lack of DA in the brain [6]. However, being a charged molecule, it cannot cross the blood–brain barrier (BBB). In spite of its crucial role in finely regulating solute passage between the circulation and the brain and serving as a rampart against pathogens, the BBB imposes substantial limitations on the range of different therapeutic interventions to address neurological medical conditions [7].

The possibility of using DA precursor levodopa instead of the neurotransmitter itself was discovered after Carlsson’s studies in the late 1950s and Cotzias’s later work [8,9]. Almost 60 years later, the use of levodopa still represents the main pharmacological approach to PD: once absorbed in the duodenum, it crosses the BBB and is converted to DA by dopaminergic neurons [10]. Other classes of drugs, such as monoamine oxidase (MAO) inhibitors, DA agonists, and catechol-O-methyltransferase inhibitors, are often used in conjunction with levodopa to prolong its half-life and sustain encephalic DA levels [11]. Nevertheless, this strategy has a constraint, as the conversion of levodopa is contingent upon the specific type of degenerating dopaminergic neurons, resulting in a gradual decline in its therapeutic efficacy in the advanced stages of the disease [12].

It follows that the ability to deliver DA directly to the CNS of PD patients would have profound beneficial effects. In recent years, numerous types of nanoparticles and therapeutic carriers, characterized by nanometer size and highly lipophilic properties, have begun to offer promising new strategies for drug incorporation and controlled delivery [13,14]. Particularly, in the context of PD, such approaches also circumvent other problems associated with the use of DA, such as its poor stability in aqueous solutions, its rapid degradation, and its inherent toxicity [15].

Therefore, the design of effective DA delivery systems for PD should meet specific criteria such as stabilizing the neurotransmitter in its natural state, avoiding cytotoxicity and dosage-related side-effects, and its release under physiological conditions. In this context, the most recently developed nanostructured devices for controlled DA release like polymeric nanoparticles and lipid-based colloidal carriers (i.e., solid lipid nanoparticles, SLNs) could represent potential alternatives to existing PD therapies [16,17,18].

From a pharmacological standpoint, attention has been given to SLNs owing to their notable attributes encompassing safety, stability, and adeptness in controlled drug release. Furthermore, they have demonstrated efficacy as carriers for diverse therapeutic agents pertinent to PD, encompassing antioxidant compounds of both polar and non-polar natures, such as curcumin, quercetin, and resveratrol [19,20,21,22,23]. 

Oxidative stress plays a significant role in PD onset, where it has been implicated in the formation of Lewy bodies or neurites—distinctive hallmarks of the disease. These structures primarily consist of aggregates of Alpha-synuclein (aS), a protein crucial for DA homeostasis and synaptic functioning [24]. Elevated levels of intracellular reactive oxygen species (ROS) appear to play a key role in compromising protein stability, ultimately leading to the generation of cytotoxic oligomeric forms and fibrils [25,26,27]. Therefore, boosting conventional drug therapies with compounds endowed with antioxidant properties has emerged as a promising pharmacological strategy for mitigating the pathological phenotype [28,29,30] serving as the starting point for our work.

In the context of a project aiming at brain delivery of new lipid nanocarriers by intranasal administration, we prepared two different types of in vitro formulations of SLNs combining the neurotransmitter DA and the natural antioxidant mixture Grape Seed Extract (GSE). We formulated SLNs in which both active principles were co-loaded with SLNs in which GSE was physically adsorbed onto preformed DA-SLNs [31,32], according to the working hypothesis that an antioxidant-based therapy might reduce ROS connected to PD along with providing a synergistic effect with the exogenous DA supply. Indeed, rather than exploiting the antioxidant effects arising from vitamin E [33,34], we selected polyphenols from GSE, mainly consisting of proanthocyanidins, to assess both their potential therapeutic and antioxidant effects.

Overall, the novelty of the present work consists of the use of a differentiated cell model derived from the SH-SY5Y cell line to evaluate the antioxidant and protective properties of DA/GSE-SLNs, as well as the change in aS protein levels, so gaining insight into the mechanisms exerted by GSE (alone or in combination with DA) beyond the direct antioxidant role. Moreover, the SH-SY5Y cell line, known for its ability to synthesize DA and replicate some characteristics of neurons, offers considerable potential for PD research while maintaining the economic benefits associated with immortalized cell lines [35,36].

## 2. Results

### 2.1. DA/GSE SLNs Show Cytobiocompatibility in SH-SY5Y-Differentiated Cell Model

**Figure 1:**
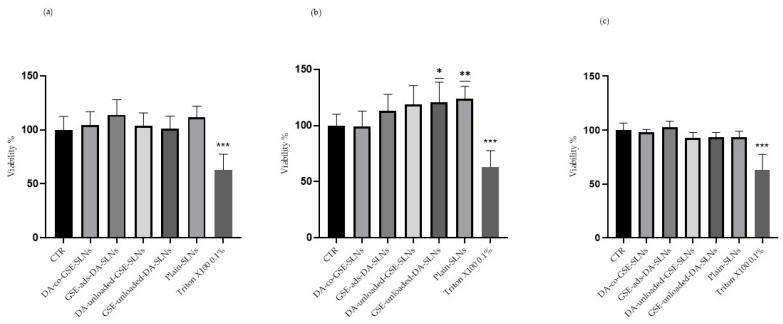
Cytocompatibility evaluation of SLNs in differentiated SH-SY5Y cells. Cells were treated with DA-co-GSE-SLNs, GSE-ads-DA-SLNs, DA-unloaded-GSE-SLNs, GSE-unloaded-DA-SLNs, and plain-SLNs at (**a**) 150 µM, (**b**) 75 µM, and (**c**) 50 µM of final concentrations of DA in growth medium at the indicated volumes in Table 2 for 24 h. A treatment with 0.1% Triton-X100 worked as positive control. Data are expressed as mean ± SD of three independent experiments replicated seven times each. Values were compared with CTR by one-way ANOVA following Dunnet test. * *p* < 0.05, ** *p* < 0.01, *** *p* < 0.0001.

The DA-co-GSE-SLNs and GSE-ads-DA-SLNs that were investigated in this study were prepared as already discussed [33,34], and some of their physicochemical properties are listed in Table 1. Regarding preliminary biological evaluation, after differentiation, cells were incubated for 24 h with SLNs to assess their cytobiocompatibility (Figure 1a–c and Figure S1, Table 2). The formulations containing DA, specifically DA-co-GSE-SLNs, GSE-ads-DA-SLNs, and GSE-unloaded-DA-SLNs, were tested at concentrations of 150 (Figure 1a), 75 (Figure 1b), and 50 µM (Figure 1c). Simultaneously, the evaluation included both SLNs, namely DA-unloaded-GSE-SLNs (containing only GSE) and plain-SLNs (devoid of both cargoes).

In all the considered conditions, SLNs did not induce notable changes in cell viability compared to the CTR, except for 75 µM GSE-unloaded-DA-SLNs and plain-SLNs (Figure 1b), which resulted in a small but statistically significant increase in cell viability. As expected, treatment with 0.1% Triton-X100 led to a significant decrease in cell viability.

### 2.2. DA/GSE-SLNs Are Cytoprotective against Rotenone-Induced Toxicity and Oxidative Stress 

To assess the potential cytoprotective effect of SLNs, Rotenone was used to induce strong intracellular stress resembling some of the pathophysiological conditions of PD.

**Figure 2:**
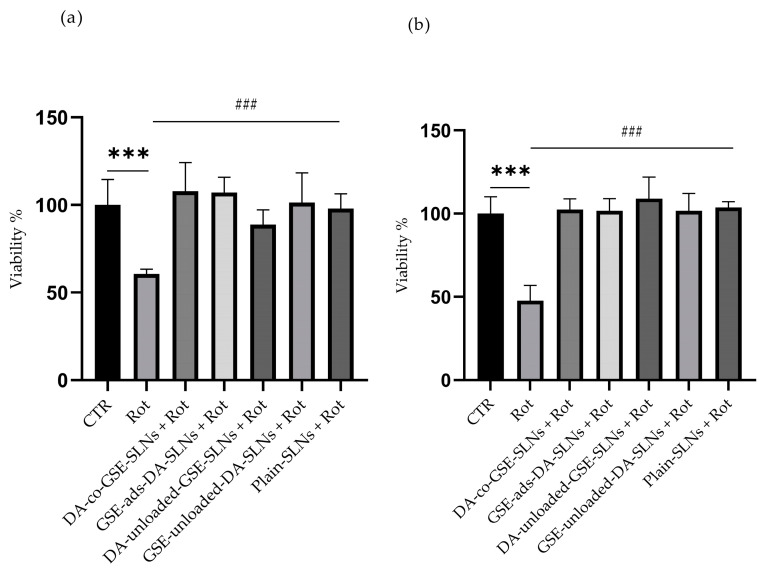
Cytoprotective effect of SLNs on differentiated SH-SY5Y cells. Cells were treated with different DA-co-GSE-SLNs, GSE-ads-DA-SLNs, DA-unloaded-GSE-SLNs, GSE-unloaded-DA-SLNs, and plain-SLNs at (**a**) 75 µM and (**b**) 50 µM final concentrations of DA in complete medium and with 5 μM Rotenone for 24 h. Rotenone-treated cells were used as positive control. Data are expressed as mean ± SD of three independent experiments replicated seven times each. Values were compared by one-way ANOVA following Dunnet test. *** *p* < 0.0001 in comparison to CTR; ### *p* < 0.0001 in comparison to Rotenone.

Hence, cells were incubated with SLNs for 24 h, and then with 5 µM Rotenone for following 24 h. Results from the MTT assay unequivocally illustrate the robust protective effects conferred by each unique preparation against the toxicant. When administered individually, Rotenone caused a significant reduction in cell viability, nearly 50% lower compared to the CTR and to the other co-treatments (Figure 2a,b).

**Figure 3:**
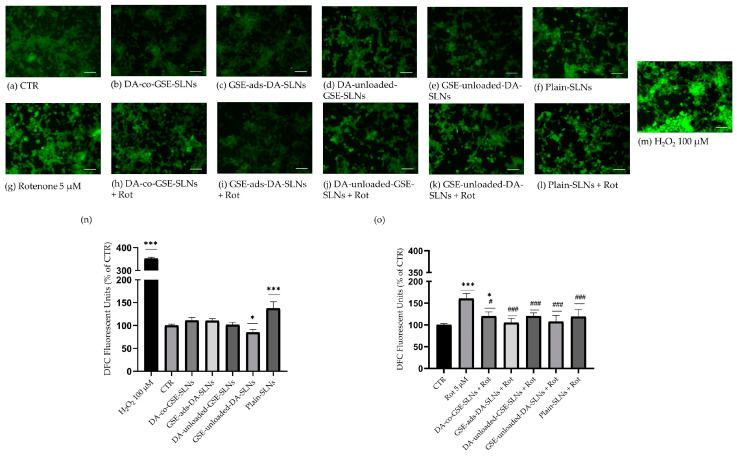
Intracellular ROS level evaluation. Representative fluorescent images of differentiated SH-SY5Y cells stained with DCFDA following a 24 h incubation with (**b**) DA-co-GSE-SLNs, (**c**) GSE-ads-DA-SLNs, (**d**) DA-unloaded-GSE-SLNs, (**e**) GSE-unloaded-DA-SLNs, and (**f**) plain-SLNs (75 µM final concentration of DA). In parallel, experiments were repeated by co-treating cells for a further 24 h with (**h**–**l**) 5 μM Rotenone. (**n**) Relative intensity of DCF in cells treated with SLNs for 24 h or (**o**) in combination with 5 µM Rotenone. Values are expressed as DCF fluorescent units and compared with (**a**) CTR, whose value was arbitrarily set as equal to 100. (**g**) Rotenone-treated cells were used as positive control, (**m**) whilst a 100 µM concentration of H_2_O_2_ was used to verify the functioning of the probe. Images are representative of two independent experiments. Data are presented as mean ± SD of two biological independent experiments with seven replicates for each experimental condition. Scale bar = 200 µm. Values were compared with CTR by one-way ANOVA following Tukey test. * *p* < 0.05, *** *p* < 0.0001 in comparison to CTR; # *p* < 0.05, ### *p* < 0.0001 in comparison to Rotenone.

In consideration of the results obtained from the previous assays, we proceeded with characterizing only the formulations of SLNs containing 75 µM DA. The antioxidant properties of SLNs underwent further investigation using the fluorescence-based DCFDA assay either in the presence or absence of Rotenone. Intracellular ROS levels were initially measured after the treatment with the different SLN formulations, without Rotenone, for 24 h (Figure 3a–f,m,n). ROS levels remained mostly unchanged compared to the CTR, except for the DA-unloaded-GSE-SLN and plain-SLN conditions. In the former case, there was a significant reduction in ROS levels compared to the CTR, whilst the treatment with plain-SLNs showed a significant increase in DCF fluorescence signal. To validate the probe function, a 100 µM H_2_O_2_ treatment was performed, showing a substantial increase in ROS levels, approximately 3.5 times that of the CTR (Figure 3m,n).

We tested the antioxidant properties of SLNs after 24 h treatment with 5 µM Rotenone (Figure 3a,g–l,o), which induced a significant increase in ROS levels exceeding 1.5 times the CTR value. In contrast, all co-treatments with Rotenone and the different nanoparticles reduced the probe fluorescence signal to CTR levels, except for a subtle but significant increase for cells pretreated with DA-co-GSE-SLN (Figure 3g,h,o). These results suggest a protective role exercised by nanoparticles against the cell oxidative stress conditions mimicked by Rotenone incubation.

### 2.3. FITC-SLNs Are Detected by Fluorescence Microscopy

**Figure 4:**
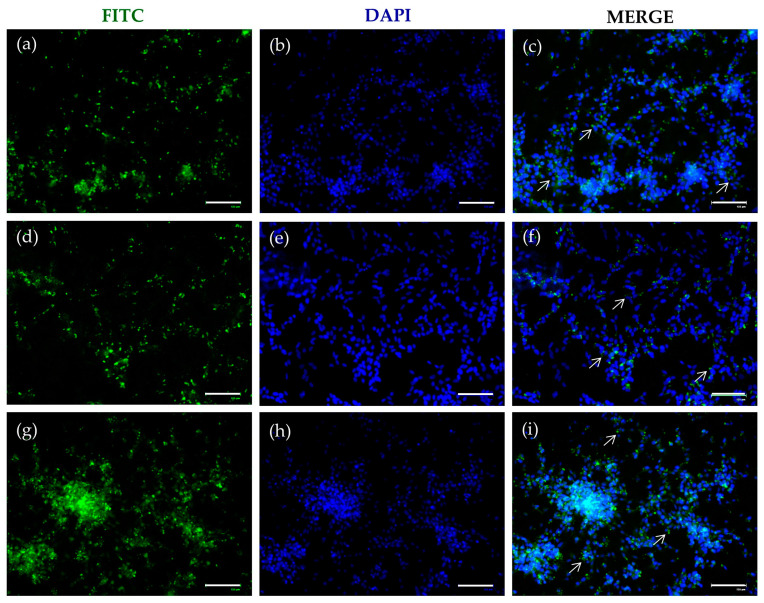
Cellular uptake of DA-FITC-SLNs coencapsulating GSE. Representative fluorescent microscopic images of differentiated SH-SY5Y cells treated with DA-FITC-SLNs coencapsulating GSE at different time points. Cells, seeded onto the coverslip, were treated with DA-FITC-SLNs coencapsulating GSE (75 µM final concentration of DA) for (**a**) 24, (**d**) 48, and (**g**) 72 h and visualized in the green channel. Cellular uptake of DA-FITC-SLNs coencapsulating GSE was imaged by DAPI in the blue channel at (**b**) 24, (**e**) 48, and (**h**) 72 h. Merged images represent the colocalization (white arrows) of DA-FITC-SLNs coencapsulating GSE and reporting nuclei after (**c**) 24, (**f**) 48, and (**i**) 72 h of treatment. Images are representative of two independent experiments. Magnification: 20×. Scale bar = 125 μm.

**Figure 5:**
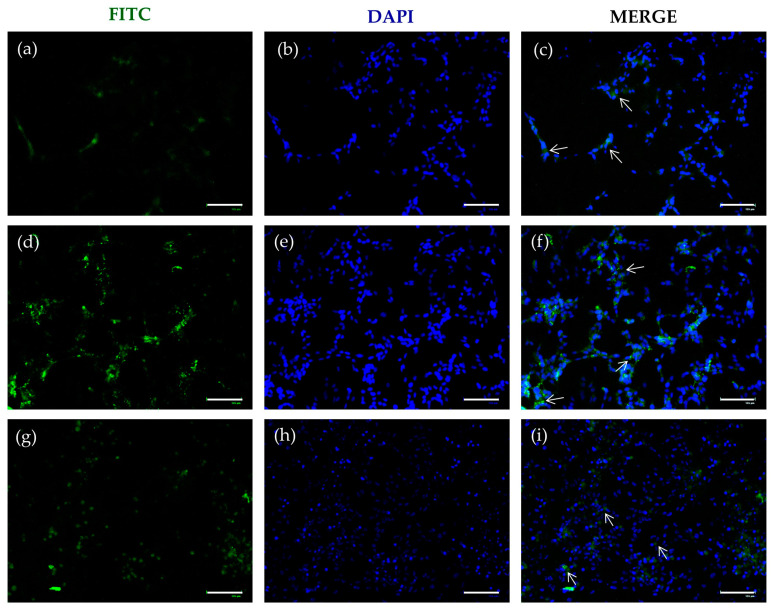
Cellular uptake of GSE-ads-DA-FITC SLNs. Representative fluorescent microscopic images of differentiated SH-SY5Y cells treated with GSE-ads-DA-FITC SLNs at different time points. Cells, seeded onto the coverslip, were treated with GSE-ads-DA-FITC SLNs (75 µM final concentration of DA) for (**a**) 24, (**d**) 48, and (**g**) 72 h and visualized in the green channel. Cellular uptake of GSE-ads-DA-FITC SLNs was imaged by DAPI in the blue channel at (**b**) 24, (**e**) 48, and (**h**) 72 h. Merged images represent the colocalization (white arrows) of GSE-ads-DA-FITC SLNs and reporting nuclei after (**c**) 24, (**f**) 48, and (**i**) 72 h of treatment. Images are representative of two independent experiments. Magnification: 20×. Scale bar = 125 μm.

The effective internalization of SLNs was assessed by replacing DA with DA-FITC loaded into the carrier. Fluorescence microscopy examination revealed a distinct green signal attributed to the fluorophore, predominantly concentrated near the nuclei, as indicated by DAPI staining. This phenomenon was consistently observed in both formulations, namely DA-FITC coencapsulating GSE (Figure 4) and GSE-ads-DA-FITC-SLN (Figure 5). This internalization process, evident already after 24 h (Figure 4a–c and Figure 5a–c) and 48 h (Figure 4d–f and Figure 5d–f) of incubation, persisted for at least 72 h (Figure 4g–i and Figure 5g–i).

The prolonged intracellular permanence of SLNs could be closely related to the biological effect observed in the previous experiments (Figure 1, Figure 2 and Figure 3).

### 2.4. DA-co-GSE-SLNs Reduce Rotenone-Mediated Increase in Intracellular aS Levels

**Figure 6:**
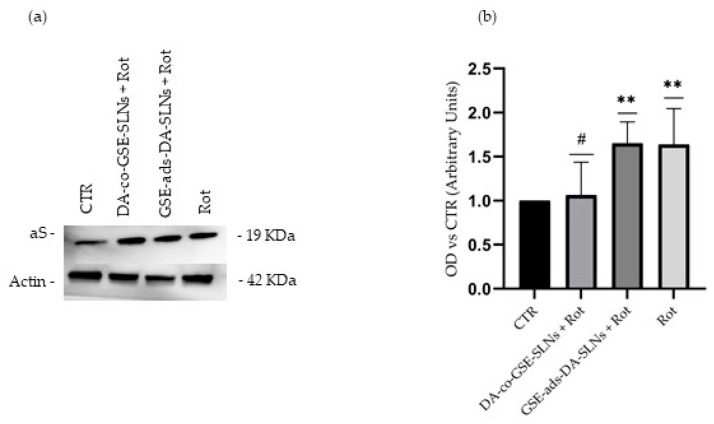
Evaluation of aS levels. (**a**) Representative immunoblots depicting aS expression. (**b**) Quantification of protein levels through densitometric analysis, presented as means ± SD from five independent experiments. Notably, a significant upregulation in aS protein levels is evident after a 24 h exposure to 5 µM Rotenone. However, this aS upregulation is significantly reduced when cells are co-incubated with Rotenone and DA-co-GSE-SLNs. In this case, aS protein levels remain close to the CTR levels, indicating a DA-co-GSE-SLN preventive effect on Rotenone-induced protein elevation. Β-actin was used as loading control. Values were compared by one-way ANOVA following Tukey’s test. ** *p* < 0.001 in comparison to CTR; # *p* < 0.05 in comparison to Rotenone.

The accumulation of aS, in vivo, appears to be a fundamental feature in PD pathology. To evaluate whether previously observed effects of SLN treatments could modulate aS levels, we conducted Western blot assays using total protein lysates from cells incubated with SLN DA-co-GSE or SLN GSE-ads-DA in co-treatment for 24 h with 5 µM Rotenone or solely with Rotenone (Figure 6a,b).

Rotenone significantly increased aS levels, a phenomenon that may be attributed to an upregulation in response to an oxidative cellular stress triggered by mitochondrial dysfunction. The DA-co-GSE-SLN formulation was able to significantly reduce the accumulation of aS protein in the presence of Rotenone, suggesting a protective antioxidant activity (Figure 6a,b).

## 3. Discussion

To date, the primary approach to pharmacological therapy in PD has mainly focused on the administration of molecules aimed at alleviating motor symptoms [6]; furthermore, it is known that aS dyshomeostasis plays an important role in both the onset and progression of PD. A promising avenue to pursue in the formulation of new therapies is represented by the synergistic effects of antioxidant and cytoprotective substances [37].

In this study, we focused on the in vitro effects of DA/GSE-SLN administration by exploiting the advantage of differentiated SH-SY5Y cells both as promising models for developing therapies for PD and for testing SLNs [38,39,40]; the same study was previously conducted on undifferentiated SH-SY5Y cells [32]. The SLNs tested here are highly performing transport systems exploited by the pharmaceutical research for their ability to efficiently cross the BBB when the particle size is in the range of 200–500 nm, so they have an increased blood circulation time and the drug is in contact with the BBB for the maximum time. Important results concerning SLNs and brain delivery were obtained when ligand-conjugated SLNs were studied [41,42,43]. For instance, ApoE-modified SLNs or transferrin-functionalized SLNs ensured the release of the therapeutic agents in a well-targeted brain district [41,42,43]. In this work, in comparison to other antioxidant agents (such as glutathione or ascorbate which is highly concentrated in the brain), GSE was our choice to address the issue of the oxidative stress connected to the neurological disorder of PD, with GSE being a mixture of different antioxidant compounds which can exert a synergistic effect.

The experimental design underlying the activities described in this article focused on evaluating two SLN formulations, namely DA-co-GSE-SLNs and GSE-ads-DA-SLNs, whose main physicochemical features are listed in Table 1. Both types of SLNs, whose size, zeta potential, and morphological external surface have been extensively characterized [31,32], were non-cytotoxic and endowed with cytoprotective effects against Rotenone activity (Figure 1 and Figure 2).

To correlate the observed biological outcomes with specific intracellular activities of SLNs, we ensured their internalization by synthesizing DA-FITC-SLN encapsulating GSE and GSE-ads-DA-FITC-SLNs. The fluorescence images depicted in Figure 4 and Figure 5 underscore the efficiency of SLNs in crossing the plasma membrane, confirming that the observed cytoprotective effects stem from an intracellular mechanism.

Rotenone, a well-known oxidant agent widely employed in creating in vitro and in vivo models of PD [44,45], induced a substantial increase in oxidative stress. However, cells pretreated with SLNs and subsequently exposed to Rotenone exhibited a significant restoration of intracellular ROS levels. Notably, GSE-ads-DA-SLNs demonstrated a greater antioxidant property compared to DA-co-GSE-SLNs (Figure 3o).

These findings are likely attributed to the presence of GSE and to its cytoprotective properties. As previously demonstrated through X-Ray Photoelectron Spectroscopy Analysis [32], the external localization of GSE was confirmed in both GSE-ads-DA-SLNs and DA-co-GSE-SLNs. However, it is plausible that in the former SLNs, the physical adsorption of the antioxidant mixture contributes more significantly to the observed cytoprotective effects.

In the literature, no report has been published describing the exposure of the antioxidant GSE to differentiated SH-SY5Y cells in the context of targeting oxidative stress related to PD. On the other hand, the neuroprotective activity of GSE against the Rotenone-induced PD model, as schematically represented in Scheme S1, was also demonstrated by the restoration of neurite loss from TH+/MAP2+ neurons and mitochondrial respiration in dopaminergic cells. This was attributed to the presence of proanthocyanidins, anthocyanins, and gallic acid in the GSE mixture [46]. The mechanism behind the effectiveness of GSE lies in its ability to interact with the mitochondrial electron transport chain. More in detail, proanthocyanidins have been demonstrated to alleviate mitochondrial dysfunction by modulating electron transport and mitigating toxicity associated with rotenone-mediated complex I inhibition. They achieve this by rescuing defects in mitochondrial respiration and restoring rotenone-induced defects in mitochondrial O_2_ consumption in neuronal cells [47]. Additionally, GSE was already found to upregulate the transcriptional co-activator PGC1α, which is involved in oxidative metabolism and mitochondrial biogenesis [48].

Finally, the treatment of SH-SY5Y cells with GSE in vitro reduced oxidative stress damage and improved antioxidant status before inducing PD with Rotenone and the neurotoxin 6-hydroxydopamine [49].

Considering the previously shown antioxidant effects of SLNs, and the recognized ability of polyphenols to safeguard the brain from oxidative damage, in the present work, we have also explored the potential of DA/GSE-SLNs as a modulator of aS accumulation.

The pathological manifestations of PD are tied to the presence of intracellular proteinaceous structures, specifically Lewy bodies and Lewy neurites, wherein the primary component is aS [50]. While the dysregulation and misfolding of aS are recognized as pivotal factors in the formation of these aggregates, it remains unclear whether these structures act as sources of cellular stress or represent protective mechanisms [51,52].

Oxidative stress, a significant factor implicated in both the initiation and progression of neurodegeneration, also plays a role in the disturbance of aS homeostasis [53].

In our study, we also assessed the potential of synthesized SLNs in preserving intracellular stability of aS.

Following Rotenone treatment, we observed a marked increase in intracellular levels of aS, suggesting a strong correlation of protein level and the oxidative cell status. Protein expression was reported as the control value when cells were exposed to 24 h of co-treatment with Rotenone and DA-co-GSE-SLNs (Figure 6a,b), also underpinning an important role for the way the antioxidant is carried inside the cell and the stability of the bond with the carrier.

Furthermore, multiple studies have established a connection between elevations in levels of aS and the disruption of the complex and extremely interconnected pathways regulating protein degradation and turn-over; this correlation, in turn, is implicated in the initiation of PD [54,55]. Such occurrences may also result from increased intracellular ROS levels [56].

Based on this assumption, recent studies have shown that the stimulation of aS degradation pathways, i.e., the ubiquitin–proteasome system and autophagy-lysosomal pathway, may represent promising approach for PD treatment [57,58]. 

Among the bioactive molecules contained in GSE, resveratrol, in addition to its antioxidant properties, has been shown to exert a remarkable role in restoring aS catabolism, by activating autophagy [59]. 

The molecule seems not only capable of inhibiting the aggregation of aS monomers but has shown some properties in disaggregating aS oligomers and fibrils [60].

Similarly, hydroxytyrosol, a natural antioxidant and ROS scavenger, together with its metabolites, has shown the capability of completely inhibiting aS fibril formation even at low doses, as well as exerting a destabilizing effect towards the aS fibrils [61].

The role of GSE in terms of aS aggregation has also been explored in in vivo models, in C57BL/6 transgenic mice overexpressing the A53T-mutant form of human aS, the first form of mutated protein strictly associated with PD onset. The GSE assumption in these mice determined a decrease in the accumulation of aS in the frontal cortex, also leading to a reduction in the expression of neuroinflammatory markers ionized calcium-binding adaptor molecule 1 (IBA1) and CD54 in both the frontal cortex and hippocampus [46].

Recently, Abdel-Salam and colleagues found how GSE has a protective effect in Rotenone-treated mice by reducing neural degeneration, apoptosis, and oxidative stress, as well as preventing functional decline [62].

Accumulating evidence suggests that GSE can induce autophagy through the PI3K/Akt/mTOR pathway, representing a promising mechanism for further investigation, also considering that the inhibition of aS accumulation is a crucial goal in PD therapy [63].

## 4. Materials and Methods

### 4.1. Materials

Grape Seed Extract (GSE) containing ≥95.0% of proanthocyanidins was received as a gift by Farmalabor (Canosa di Puglia, Italy). Gelucire^®^ 50/13 was kindly donated by Gattefossé (Milan, Italy). Dopamine hydrochloride, fluorescein 5(6)-isothiocyanate (FITC), Tween^®^ 85, carboxyl ester hydrolase (esterases, E.C. 3.1.1.1, 15 units/mg powder), as well as the salts used for buffer preparation were bought from Sigma-Aldrich (Milan, Italy). Dopamine–fluorescein isothiocyanate (DA-FITC) was synthesized by following the procedure mentioned in Carta et al.’s work [64]. In this work, double-distilled water was used, and all other chemicals were of reagent grade.

### 4.2. DA/GSE-SLN Preparation

Two main types of SLNs were tested, DA-coencapsulating GSE and GSE-adsorbing DA-SLNs.

(i) *DA-coencapsulating GSE SLNs (DA-co-GSE-SLNs).* The formulation “DA-co-GSE-SLNs” was obtained following the melt homogenization method [49,65]. An amount of 60 mg of Gelucire^®^ 50/13 was melted at 70 °C and, separately, 6 mg of GSE was dispersed in the aqueous phase made of surfactant (Tween^®^ 85, 60 mg) and 1.37 mL diluted acetic acid (0.01%, *w*/*v*). The predispersion was homogenized at 12,300 rpm with an Ultra-Turrax model T25 apparatus (Janke and Kunkel, IKA^®^-Werke GmbH & Co., Staufen, Germany) and allowed to equilibrate for 30 min at 70 °C. Then, 10 mg of DA was introduced in the aqueous phase, the resulting mixture was added to the melted phase at 70 °C, and the so-obtained emulsion was homogenized at 12,300 rpm for 2 min by the Ultra-Turrax system. Then, the nanosuspension was cooled at room temperature and allowed to achieve SLN DA-coencapsulating GSE. Such SLNs were centrifuged (16,000× *g*, 45 min, Eppendorf 5415D, Hamburg, Germany) and the pellet was harvested and re-suspended in distilled water for further studies, but the supernatant was discarded.
(ii) *GSEadsorbing DA-SLNs (GSE-ads-DA-SLNs).* For DA SLNs adsorbing GSE (called GSE-ads-DA-SLNs), DA-loaded SLNs were prepared as previously described [32], but starting from 20 mg of DA rather than 10 mg to force DA initial *cargo*. After cooling down at room temperature, an aliquot of 0.5 mL of the resulting DA-SLNs was incubated with 1 mL of GSE aqueous solution (1 mg/mL concentration) at room temperature for 3 h in the dark while maintaining mild stirring (50 oscillations/min). Then, the mixture was centrifuged at 16,000× *g* for 45 min (Eppendorf 5415D) and the pellet was dispersed in distilled water, whereas the supernatant was discarded. For comparison, the following formulations were also tested in the biological assays: SLNs loaded with DA only, formulated without GSE (called “GSE-unloaded-DA-SLNs”), and SLNs containing GSE without DA (called “DA-unloaded-GSE-SLNs”).
(iii) As the control, SLNs with neither DA nor GSE were formulated following the melt homogenization method as reported above and are indicated as plain-SLNs in this paper. For the fluorescence microscopy application, two types of fluorescent SLN carriers were obtained as follows. Firstly, to achieve the fluorescent “DA-FITC-SLNs coencapsulating GSE”, 10 mg of DA-FITC replaced 10 mg of DA and the protocol was the same as that shown in Section 2.2. (i). Another fluorescent formulation of SLNs was produced by adsorbing 1 mL of GSE aqueous solution (1 mg/mL) onto 0.5 mL of preformed DA-FITC-SLNs. The physical adsorption was carried out for 3 h at room temperature and in the dark, leading to the formation of SLNs called “GSE-ads-DA-FITC SLNs”.

### 4.3. In Vitro Properties of DA/GSE SLNs

For the mean particle size and polydispersity index (PDI) of the SLNs (Table 1), Photon Correlation Spectroscopy (PCS) of the ZetasizerNanoZS (ZEN 3600, Malvern, UK) apparatus was adopted. Samples were located in folded capillary zeta cells with a measurement angle of 173° Backscatter. The particle size and PDI values of the SLNs were measured at 25 °C by redispersing each sample in 1 mL of double-distilled water after a short sonication. Then, the nanosuspensions underwent a further dilution in double-distilled water (1:200, *v*/*v*) prior to analysis for particle size. For ζ-potential measurements, the laser Doppler anemometry technique was adopted (ZetasizerNanoZS, ZEN 3600, Malvern, UK) by using the same dilution employed for size analysis.

Moreover, the HPLC apparatus allowed the quantification of DA and GSE, according to procedures previously reported with slight modifications [66,67]. Briefly, the HPLC station included a Waters Model 600 pump (Waters Corp., Milford, MA, USA), a Waters 2996 photodiode array detector, and a 20 μL loop injection autosampler (Waters 717 plus). The stationary phase, Synergy Hydro-RP (25 cm × 4.6 mm, 4 μm particles; Phenomenex, Torrance, CA, USA), was eluted with a 0.02 M potassium phosphate buffer, pH 2.8: CH_3_OH 70:30 (*v*/*v*), as a mobile phase in isocratic mode at the flow rate of 0.7 mL/min. Under such chromatographic conditions, the retention times of DA and GSE were found to be equal to 5.5 min and 12 min, respectively.

For DA and GSE quantification, the enzymatic digestion of SLNs by esterases was carried out by dissolving the enzyme at 12 I.U./mL in phosphate buffer (pH 5) and 1–2 mg of freeze-dried SLNs was incubated with 1 mL of the enzyme solution for 30 min in an agitated (40 rpm/min) water bath set at 37 °C (Julabo, Milan, Italy). Then, the resulting mixture was centrifuged (16,000× *g*, 45 min, Eppendorf 5415D) and the supernatant was analyzed via HPLC analysis, as above.

For standardization of FITC, the calibration of the fluorometer (Perkin Elmer, Milan, Italy) adopted solutions ranging from 1 to 140 ng/mL of FITC in phosphate buffer, pH 8.0, obtained from the dilution in such a buffer of a previously prepared stock solution of 100 μg/mL FITC in methanol. For fluorometric analysis and excitation, emission wavelengths of 488 and 525 nm, respectively, were selected, and slits were set at 2.5 cm.

The association efficiency (A.E.%) was calculated by Equation (1):

A.E.% = DA (GSE or FITC) in the supernatant after esterase assay/Total DA (GSE or FITC) × 100(1)

where the total DA (GSE or FITC) is the starting amount of each substance used for SLN preparation. The study was performed in triplicate.

### 4.4. Cell Culture and Treatments

The Human neuroblastoma SH-SY5Y cell line was purchased from the European Collection of Cell Culture (ECCC, Salisbury, UK) and grown in high-glucose Dulbecco’s Modified Eagle Medium (DMEM) (Sigma, St. Louis, MO, USA) supplemented with 1 mM sodium pyruvate (Gibco, New York, NY, USA) and 10% (*v*/*v*) fetal bovine serum (FBS) (Sigma) in an incubator, at 37 °C in a 5% CO_2_ atmosphere.

Neuronal differentiation was induced as previously described [68]. In brief, cells were initially seeded at a density of 3 × 10^4^ cells/cm^2^ and cultured with the addition of 10 µM Retinoic Acid (RA) (Sigma) and 2% fetal bovine serum (FBS) for the first 6 days. Subsequently, the culture conditions were modified to include 10 µM RA (Sigma) and 1% (*v*/*v*) FBS for the next 3 days. In the final 5 days of the differentiation protocol, cells were maintained in Neurobasal medium (Gibco) supplemented with 50 ng/mL of Brain-Derived Neurotrophic Factor (BDNF) (PeproTech, Rocky Hill, NJ, USA) and 1× B27 (Gibco).

Cells underwent a 24 h treatment with various formulations of DA/GSE SLNs, encompassing DA-unloaded-GSE-SLNs and GSE-unloaded-DA-SLNs, as detailed in Table 2. The chosen concentrations for DA were 150, 75, and 50 μM. In the case of plain-SLNs, their volumes were adjusted to ensure an equivalent lipid content to that of the other formulations.

After conducting cytobiocompatibility experiments with SLNs alone, subsequent investigations included a 24 h cell pretreatment with various SLN formulations, followed by a 24 h treatment with 5 µM Rotenone (Sigma).

### 4.5. DA/GSE-SLN Cytobiocompatibility Evaluation

Prior to evaluating the potential beneficial effects present in various SLN preparations, an assessment of their cytobiocompatibility in the cell model was conducted.

Cells were seeded in a 96-well plate (Becton, Dickinson and Company, Franklin Lakes, NJ, USA) and incubated for 24 h as previously described.

The medium was then substituted with a 0.5 mg/mL solution of 3-(4,5-dimethylthiazol-2-yl)-2,5-diphenyltetrazolium bromide (MTT) (Sigma) in fresh medium and incubated for 1 h. Cellular lysis was achieved through the addition of dimethyl sulfoxide (DMSO).

The colorimetric assay relies on the cleavage of tetrazolium salts introduced into the culture medium. Enzymes from the endoplasmic reticulum catalyze this process, leading to the formation of formazan. This bioreduction specifically occurs in viable cells and is linked to NAD(P)H production through glycolysis. Consequently, the amount of formazan dye formed serves as a direct indicator of the number of metabolically active cells in the culture [69].

The quantification of absorbance within the samples occurred at a wavelength of 595 nm, utilizing a Multiskan™ FC Microplate Photometer (Thermo Fisher Scientific, Waltham, MA, USA). A subsequent measurement at 620 nm was undertaken for the purpose of background subtraction. The impact of each distinct treatment was normalized in reference to the untreated control (CTR), a baseline value arbitrarily established at 100. Notably, a treatment of select wells with 0.1% (*v*/*v*) Triton-X100 served as a positive control for the entirety of the experimental investigation.

### 4.6. Evaluation of DA/GSE-SLN Cytoprotective Effects 

Using the same method as described above, the cytoprotective effect of the different SLN formulations was evaluated following a 24 h incubation and after a further 24 h of treatment with 5 µM Rot. Cells subjected solely to a 5 μM Rotenone exposition for a duration of 24 h worked as the positive control. Also in this case, obtained values were normalized in reference to the CTR.

### 4.7. FITC-DA/GSE-SLN Fluorescence Microscopy 

Cells were seeded and grown on coverslips adherent to the bottom of a 12-well plate and incubated for 24, 48, or 72 h with 75 µM DA-FITC-SLNs coencapsulating GSE or GSE-ads-DA-FITC-SLNs. After fixation with 4% (*v*/*v*) paraformaldehyde in PBS for 15 min at room temperature, cells were permeabilized in PBS containing 0.05% Triton-X100 for 10 min and rinsed with PBS twice. Coverslips were counterstained and mounted on slides using a mounting medium with DAPI (Fluoromount-G™ Mounting Medium) (Thermo Fisher Scientific) and visualized with an EVOS Floid fluorescence microscope (Thermo Fisher Scientific).

### 4.8. DA/GSE-SLN Antioxidant Property Assay

The intracellular ROS content in the presence of the different experimental conditions was estimated using the 2′,7′-dichlorofluorescin diacetate (DCFDA) fluorescent probe (Sigma). The operational principle of the assay hinges on the cleavage of a specific molecule within the cellular environment by esterases. This enzymatic activity leads to the generation of the product H_2_DCF, which remains confined within the cell due to its membrane impermeability property. Subsequently, an oxidative process occurs, proportionate to the concentration of hydrogen peroxide within the cellular milieu, resulting in the formation of an intensely fluorescent product known as DCF. The resultant fluorescence is directly proportional to the quantity of DCFDA oxidized to DCF and can be quantified in the green channel at a wavelength of 529 nm. Consequently, the redox state of the sample is effectively monitored by the detection of the observed increase in fluorescence intensity [70,71].

Following treatments, cells were incubated for 30 min with fresh medium containing DCFDA, and then washed with PBS and analyzed with a Cytation 5 Imaging plate reader (Biotek Instruments Inc., Agilent, CA, USA) (Ex/Em 485/535 nm). The obtained fluorescence was directly proportional to the amount of oxidized DCFDA to DCF.

### 4.9. Western Blotting

Immunoelectrophoresis was performed on cell extracts obtained by treating cells with ice-cold RIPA buffer (Cell Signalling Technologies, Danvers, MA, USA) and centrifuged at 12,000 rpm for 20 min at 4 °C. Protein extracts were quantified with Bradford’s method and approximately 25 µg of the lysate was loaded on 14% SDS–PAGE gels. 

After the electrophoretic separation, proteins were transferred onto a PVDF membrane (Amersham Protean, GE Healthcare, Chalfont Buckinghamshire, UK). The membrane was blocked in Blotto A (Santa Cruz Biotechnology, Dallas, TX, USA) for 1 h, rinsed, and incubated with a primary anti-aS antibody (rabbit mAb, 1:1000) (Novus, Minneapolis, MN, USA) overnight at 4 °C. After discarding the antibody, membranes were washed with Tris-buffered saline (TBS) and 0.05% Tween-20 three times and then incubated with a proper HRP-conjugated secondary anti-rabbit antibody (goat mAb, 1:50,000) (Thermo Fisher Scientific) for 1 h at room temperature. After three further washes, protein–antibody complexes were visualized with the Clarity^TM^ Western ECL Substrate (Bio-Rad Laboratories, Hercules, CA, USA) and ChemiDoc^TM^ Imaging System (Bio-Rad Laboratories). Densitometric analyses were performed using the open-source software ImageJ 1.48v (https://imagej.net/software/imagej, accessed on 28 September 2021) [72] to determine the optical density (OD) of the bands. 

The protein expression was normalized to β-actin to account for variations in loading. An HRP-conjugated anti-β-actin antibody (mouse mAb, 1:25,000) (Sigma) was used for this purpose.

### 4.10. Statistical Analysis

For physicochemical data, mean ± SD and statistical evaluation were obtained using Prism v. 5.0 (GraphPad Software Inc., La Jolla, CA, USA). For biological evaluation, the results are presented as mean ± SD from at least three biological replicates. Multiple comparisons were based on one-way analysis of variance (ANOVA) with the Dunnet’s post hoc test. Statistical analyses were performed by using Prism v. 8.0.1 software (GraphPad Software Inc., La Jolla, CA, USA) and a value of *p* < 0.05 was accepted as the level of significance. The following statistical representations are used in the text: * *p* < 0.05, ** *p* < 0.01, *** *p* < 0.001, # *p* < 0.05, ### *p* < 0.001.

## 5. Conclusions

In summary, our research proposes that DA/GSE-SLNs could serve as a safe, efficient, and promising delivery system for DA. These SLNs enhance the traditional therapeutic strategy by incorporating the neuroprotective and antioxidant attributes of GSE, thereby enabling the formulation to traverse the BBB. These innovative approaches have the potential to enhance the delivery of DA and GSE, together with non-cytotoxic and cytoprotective roles against Rotenone, while concurrently alleviating potential adverse effects. Moreover, the ability of these carriers to modulate aS levels in cells positions them as significant contributors in the treatment of PD. Overall, future perspectives of our work will be addressed to test DA/GSE-SLNs with selected animal models to validate the feasibility of the nose-to-brain delivery approach.

## Figures and Tables

**Table 1:** Physicochemical properties of SLNs. Mean ± SD of at least eight replicates is reported.

Formulation	Size (nm)	PDI ^a^	Zeta Potential (mV)	A.E. DA (%)	A.E. GSE (%)	A.E. FITC (%)
DA-co-GSE-SLNs	187 ± 4 **	0.49 ± 0.04	−4.1 ± 0.1 **	62 ± 4	10 ± 0	-
GSE-ads-DA-SLNs	287 ± 15 **	0.53 ± 0.01	−7.8 ± 0.4 **	65 ± 6	57 ± 8	-
DA-FITC-co-GSE-SLNs	297 ± 25 **	0.59 ± 0.04	−3.6 ± 0.1 **	82 ± 4	23 ± 1	94 ± 6
GSE-ads-DA-FITC-SLNs	266 ± 12 **	0.57± 0.04	−8.1 ± 0.1 **	35 ± 1	42 ± 2	96 ± 1
Plain-SLNs ^b^	141 ± 11	0.35 ± 0.17	−9.7 ± 0.8	-	-	-

For statistical evaluation, plain-SLNs were controls. ^a^ PDI: polydispersity index. ^b^: From Reference [32]. ** *p* ≤ 0.001.

**Table 2:** Volumes of SLNs (µL/mL), DA concentrations (µM), and GSE amounts (µg/mL) used in the treatments.

	CTR			DA-co-GSE-SLNs			GSE-ads-DA-SLNs			DA-Unloaded-GSE-SLNs			GSE-Unloaded-DA-SLNs			Plain-SLNs		
SLN volume (µL)	-	-	-	30	15	10	30	15	10	30	15	10	30	15	10	30	15	10
[DA] (µM)	-	-	-	150	75	50	150	75	50	-	-	-	150	75	50	-	-	-
GSE amount (µg/mL)	-	-	-	12	6	4	0.02	0.01	0.0067	12.6	6.3	4.2	-	-	-	-	-	-

## Data Availability

The datasets generated during the current study are available from the corresponding authors on reasonable request.

## Supplementary Materials

The following supporting information can be downloaded at https://www.mdpi.com/article/10.3390/molecules29081774/s1. Figure S1: Phase contrast images of cellular morphology of differentiated SH-SY5Y cells after (b,h,n) DA-co-GSE-SLNs, (c,i,o) GSE-ads-DA-SLNs, (d,j,p) DA-unloaded-GSE-SLNs, (e,k,q) GSE-unloaded-DA-SLNs, (f,l,r) plain-SLNs, and (s) TRITON X-100 treatments at the indicated volumes and DA concentrations for 24 h. (a,g,m) Differentiated SH-SY5Y cells without treatments. Magnification: 20×. Scale bar = 100 µm. Scheme S1: Scheme of GSE-SLN cellular pathway. The image was created with BioRender.com.
