# Supplementary material for: Dopamine- and Grape-Seed-Extract-Loaded Solid Lipid Nanoparticles: Interaction Studies between Particles and Differentiated SH-SY5Y Neuronal Cell Model of Parkinson’s Disease
Source: Molecules. 2024 Apr 13;29(8):1774. doi: 10.3390/molecules29081774 (PMC11051794; doi:10.3390/molecules29081774)
Supplement: Supplementary file 1 [file molecules-29-01774-s001.zip › molecules-2929449-supplementary.pdf]

molecules

MDPI

Supplementary Materials

Dopamine- and Grape-Seed-Extract-Loaded Solid Lipid Nanoparticles: Interaction Studies between Particles and Differentiated SH-SY5Y Neuronal Cell Model of Parkinson's Disease

Rosanna Mallamaci [1,†], Debora Musarò [2,†], Marco Greco [2], Antonello Caponio [3], Stefano Castellani [4], Anas Munir [5], Lorenzo Guerra [1], Marina Damato [6], Giuseppe Fracchiolla [3], Chiara Coppola [5], Rosa Angela Cardone [1], Mehdi Rashidi [5], Roberta Tardugno [3], Sara Sergio [2], Adriana Trapani [3,*] and Michele Maffia [6,*]

[1] Department of Biosciences, Biotechnologies and Environment, University of Bari "Aldo Moro", 70125 Bari, Italy; rosanna.mallamaci@uniba.it (R.M.); lorenzo.guerra1@uniba.it (L.G.); rosaangela.cardone@uniba.it (R.A.C.)
[2] Department of Biological and Environmental Science and Technology, University of Salento, Via Lecce—Monteroni, 73100 Lecce, Italy; debora.musaro@unisalento.it (D.M.); sara.sergio@unisalento.it (S.S.)
[3] Department of Pharmacy-Drug Sciences, University of Bari "Aldo Moro", Via Orabona 4, 70125 Bari, Italy; antonello.caponio@gmail.com or antonello.caponio@uniba.it (A.C.); giuseppe.fracchiolla@uniba.it (G.F.); roberta.tardugno@uniba.it (R.T.)
[4] Department of Precision and Regenerative Medicine and Ionian Area (DiMePRe-J), University of Bari "Aldo Moro", 70125 Bari, Italy; stefano.castellani@uniba.it
[5] Department of Mathematics and Physics "E. De Giorgi", University of Salento, Via Lecce—Arnesano, 73100 Lecce, Italy; chiara.coppola@unisalento.it (C.C.); mehdi.rashidi@unisalento.it (M.R.)
[6] Department of Experimental Medicine, University of Salento, Via Lecce—Monteroni, 73100 Lecce, Italy; marina.damato@unisalento.it
* Correspondence: adriana.trapani@uniba.it (A.T.); michele.maffia@unisalento.it (M.M.); Tel.: +39-0805-442114 (A.T.); +39-0832-298670 (M.M.)
† These authors have contributed equally to this work.

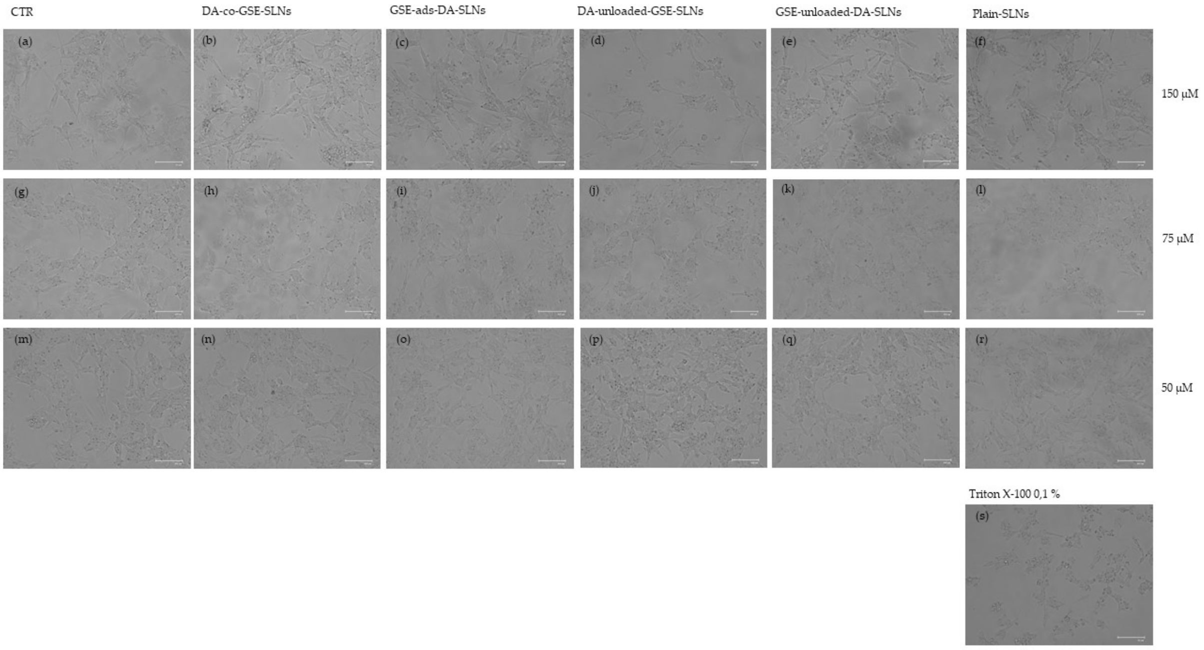

Figure S1. Phase contrast images of cellular morphology of differentiated SH-SY5Y cells after (**b, h, n**) DA-co-GSE-SLNs, (**c, i, o**) GSE-ads-DA-SLNs, (**d, j, p**) DA-unloaded-GSE-SLNs, (**e, k, q**) GSE-unloaded-DA-SLNs, (**f, l, r**) plain-SLNs and (**s**) TRITON X-100 treatments at the indicated volumes and DA concentrations for 24 hours. (**a, g, m**) Differentiated SH-SY5Y cells without treatments. Magnification: 20 X. Scala bar = 100 μm.

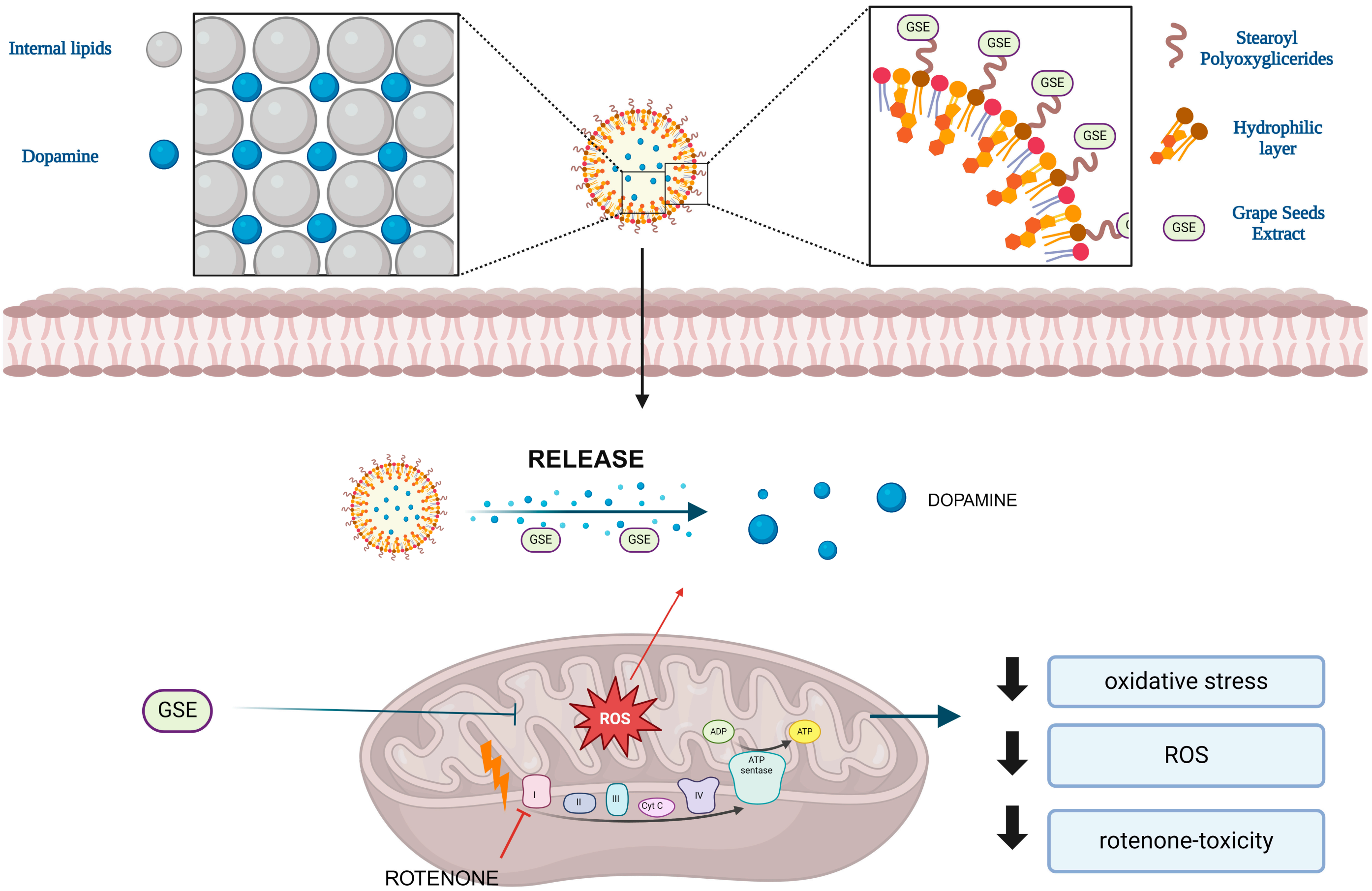

Scheme S1. Scheme of GSE-SLNs cellular pathway. The image was created with BioRender.com.
